# Towards understanding the presence/absence of Human African Trypanosomosis in a focus of Côte d'Ivoire: a spatial analysis of the pathogenic system

**DOI:** 10.1186/1476-072X-4-27

**Published:** 2005-11-03

**Authors:** Fabrice Courtin, Vincent Jamonneau, Emmanuel Oké, Bamoro Coulibaly, Yohan Oswald, Sophie Dupont, Gérard Cuny, Jean-Pierre Doumenge, Philippe Solano

**Affiliations:** 1 Institut Pierre Richet (IPR), équipe « THA et glossines », s/c IRD, Rue Fleming zone 4C, 04 BP 293, Abidjan 04, Côte d'Ivoire; 2 Institut de Recherche pour le Développement (IRD), Unité de Recherche UR 177, Laboratoire de Recherche et de Coordination sur les Trypanosomoses (LRCT IRD-CIRAD), TA 207/G, Campus International de Baillarguet, 34398 Montpellier cedex 5, France; 3 University of Lille, USTL/LGMA (Laboratoire de Géographie des Milieux Anthropisés), UMR CNRS 8141, France; 4 University of Montpellier 3, UFR sciences humaines et sciences de l'environnement, route de Mende 34199 Montpellier Cedex 5, Laboratoire de recherche GESTER (gestion des territoires et des risques), France

## Abstract

**Background:**

This study aimed at identifying factors influencing the development of Human African Trypanosomosis (HAT, or sleeping sickness) in the focus of Bonon, located in the mesophile forest of Côte d'Ivoire. A previous study mapping the main daytime activity sites of 96 patients revealed an important disparity between the area south of the town- where all the patients lived- and the area north of the town, apparently free of disease. In order to explain this disparity, we carried out a spatial analysis of the key components of the pathogenic system, i.e. the human host, the tsetse vector and the trypanosomes in their environment using a geographic information system (GIS).

**Results:**

This approach at the scale of a HAT focus enabled us to identify spatial patterns which linked to the transmission and the dissemination of this disease. The history of human settlement (with the rural northern area exploited much earlier than the southern one) appears to be a major factor which determines the land use pattern, which itself may account for differences found in vector densities (tsetse were found six times more abundant in the southern rural area than in the northern). Vector density, according to the human and environmental context in which it is found (here an intense mobility between the town of Bonon and the rural areas), may explain the observed spatial differences in HAT prevalence.

**Conclusion:**

This work demonstrates the role of GIS analyses of key components of the pathogenic system in providing a better understanding of transmission and dissemination of HAT. Moreover, following the identification of the most active transmission areas, and of an area unfavourable to HAT transmission, this study more precisely delineates the boundaries of the Bonon focus. As a follow-up, targeted tsetse control activities starting north of Bonon (with few chances of reinvasion due to very low densities) going south, and additional medical surveys in the south will be proposed to the Ivoirian HAT control program to enhance the control of the disease in this focus. This work also shows the evolution of HAT regarding time and environment, and the methodology used may be able to predict possible sleeping sickness development/extinction in areas with similar history and space organization.

## Background

Human African Trypanosomosis (HAT) or sleeping sickness, is a vector-borne parasitic disease of Sub-Saharan Africa. The parasite (*Trypanosoma brucei gambiense *in West and Central Africa) is transmitted to humans by the bite of a dipteran insect, the tsetse fly (*Glossina sp*.). The disease, though almost eradicated in the early 60's, has once again become a major public health problem. Currently, about 60 million people are at risk of infection and around 300,000 are estimated to have the disease [1]. Two principal parameters are usually put forward to explain this resurgence. First, the routine measures that were implemented to control the disease have gradually disappeared [2]. Secondly, changes in the environment, through their effect on the relationships between host, vector and parasite, may also account for a significant part of the disease's re-emergence [3]. In the forest area of Côte d'Ivoire, sleeping sickness has usually been associated with coffee and cocoa plantations [4]. The establishment of these cash crop plantations, combined with massive immigration of agricultural labour, has altered the original habitat and caused the disappearance of the mainly zoophilic forest tsetse fly species, which have been replaced by vectors with a more opportunistic feeding pattern such as the main vector of sleeping sickness *G. palpalis*, that are able to adapt to peri-urban or urban areas [5-8]. The rise in numbers of agricultural workers also led to increased vector-host contact [9]. The establishment of new villages (defined as inhabited by several families with a chief) and more especially new encampments (defined as inhabited by one family or by agricultural labourers, in coffee/cocoa plantations) also increased levels of movement along the new communication routes, further increasing human-vector contact [10-12]. Though such change is now widely accepted as a major cause of the development of sleeping sickness in the forest area of West Africa, it has yet to be discovered why the disease is present in some places but not in others, where demographic, behavioural and environmental conditions appear to be similar.

**Figure 1:**
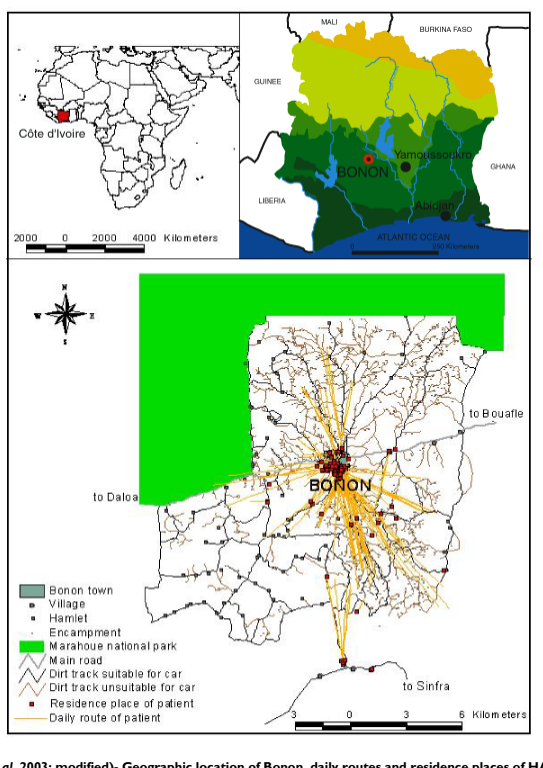
**(Solano *et al*. 2003; modified)- Geographic location of Bonon, daily routes and residence places of HAT patients**. The upper maps show the locations of Côte d'Ivoire in Africa and the focus of Bonon in Côte d'Ivoire. The bottom map shows the living places and the mobility of patients. The majority of patients live in or move to the southern rural area, whereas the northern rural is almost free of disease.

The focus of Bonon is located in the mesophile forest of Côte d'Ivoire (figure 1). The evolution of HAT prevalence in this focus since the 1950s (data supplied by the HAT National Control Program), enables to trace back the appearance and the spatial evolution of disease. The disease has been endemic for a long time with 9 cases being detected between 1956 and 1959, 57 between 1976 and 1985, 102 between 1986 and 1996, then 16 in the year 1997. These patients came principally from the south rural area. Since then 3 medical surveys in 1998 and 1999 detected a further 33 cases from visits to 7,824 people also principally from the south rural area. The following year, an exhaustive medical survey was undertaken in the area during which 15 000 people were visited and 74 cases detected, none of these cases living in the north rural area. Previous work has mapped the places of residence, water supply sites and day time activity of 96 patients who were diagnosed to have sleeping sickness. This mapping revealed an important disparity between the rural area south of the town and the rural area north of the town: the southern rural area was very affected by HAT, as shown by a high prevalence, and a high rate of activity by urban patients. By contrast, the northern rural area was characterized by a low prevalence and by a very low attendance of urban patients (figure 1). This disparity allowed to identify different areas where the mechanisms of HAT transmission may differ [11]. The aim of the present work was thus to carry out a spatial analysis of key components of the pathogenic system, taking into account parasitological, entomological, human and environmental factors, in order to try to explain the appearance and the maintenance of HAT in the southern area of this focus and its absence in the northern area. The work was conducted in four steps: (1) a study of the relationship between tsetse fly distribution and the daily activity patterns of patients, (2) a study of the history of human settlement in the focus, including the relationships between the different ethnic groups and observed land use patterns, (3) a detailed description of the land cover and land use of the area using a ground transect approach, combined with remote sensing analyses, and (4) the characterization of spatial human mobility patterns, particularly between the town of Bonon and its surroundings.

## Results

### Entomological survey

**Figure 2:**
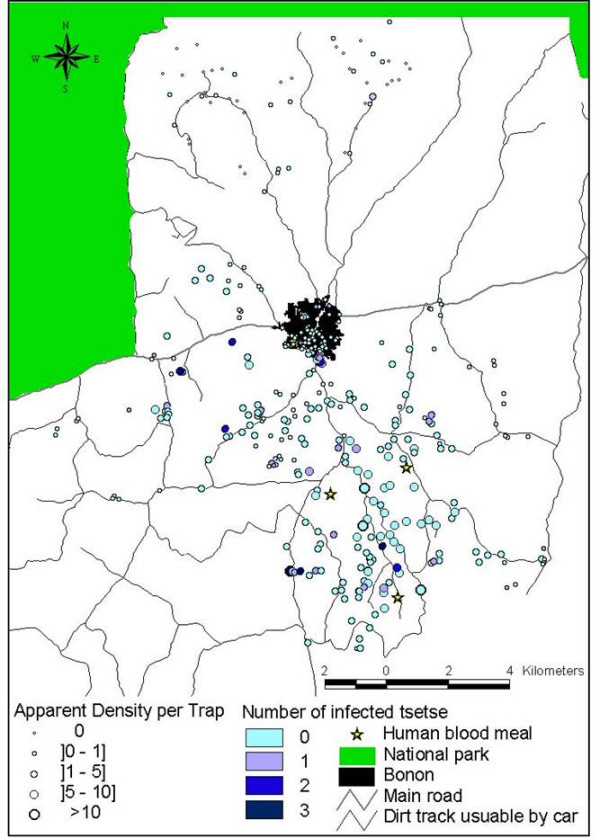
**Density, infection and human bloodmeals of tsetse flies**. This map shows the results of entomological survey. Tsetse flies are far more abundant in the southern rural area than in the northern rural area. Some tsetse flies were caught in the town. All the *T. brucei *infected tsetse flies were caught in the southern rural area and in the town, except one in the northern rural area. All the bloodmeals taken on humans by tsetse flies were located in the south rural area.

Results of the entomological survey are shown in Figure 2. In the northern rural area, out of 67 traps, the mean Apparent Density of Tsetse flies (ADT) was 0.62 tsetse/trap/day. In the southern rural area, out of 223 traps mean ADT was substantially higher, at 4.2 tsetse/trap/day. Differences in the number of traps reflect the number of patients in each of the two areas since traps were placed at their main sites of activity. In the northern rural area, out of 51 tsetse flies dissected, one was found infected with *Trypanosoma brucei *(infection rate 0.197 with Standard Deviation 0.14). In the southern rural area, out of 1496 tsetse flies dissected, 40 were infected (infection rate 0.027 with Standard Deviation 0.15). In the northern rural area, no human blood meal was found out of 5 analyzed blood meals. In the southern rural area, three human blood meals were found out of 87 analyzed blood meals.

### Geographical survey

#### History of settlement

**Figure 3:**
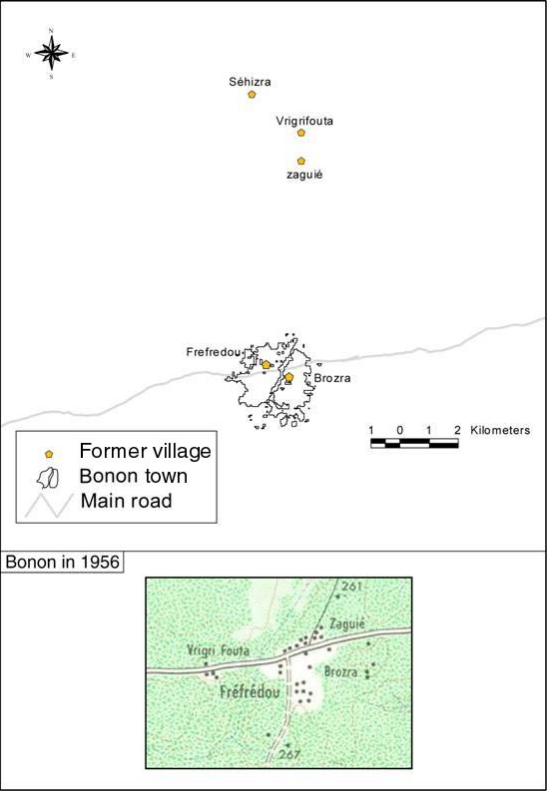
**Location of former villages and Bonon in 1956**. The upper map shows the former villages in the north, displaced during the french colonisation in the 1920s. The bottom image shows Bonon in 1956, with the former villages of the North that have been moved near the main road.

Before the present town of Bonon was created, the people of the area used to live in 5 distinct villages. The villages of Brozra and Frefredou were located on the present site of the town, near the main road (figure 3) and now constitute districts of Bonon. At the time of colonization, there were not enough people in Brozra and Frefredou to need colonial administration. It was then decided to create a large settlement by moving the inhabitants of the three villages located several kilometers to the north (Séhizra, Vrigrifouta, Zaguié) close to the main road. The resistance of villagers refusing to leave their lands, forced the government to burn the villages in the 1920s, after which the inhabitants settled next to Brozra and Frefredou villages as shown on the topographic map made by the French « Institut de Géographie National » (IGN), from aerial photography taken in 1956 (figure 3). The first non-native ethnic group (Malinke) arrived at the beginning of the twentieth century mainly to trade cola nuts. At that time, the original population was living mainly from hunting and from the cultivation of cash crops (rice and banana). During colonial period, the French government forcibly displaced large numbers of people from Upper Volta (principally the Mossi ethnic group) to work in Côte d'Ivoire, to provide labour for the development of the colony's railways and roads [14]. In 1936, the colonial administration created "villages of colonisation" to house part of the agricultural labour force (Mossi ethnic group) needed to develop the coffee and cocoa plantations in the Central West part of Côte d'Ivoire [15]. With the end of involuntary work (Félix Houphouët-Boigny law, 1946), agricultural laborors were allowed to travel freely. Since 1950, the development of cash crops (cocoa, coffee) in the centre-western region of Côte d'Ivoire (Vavoua, Daloa, Bouaflé, Gagnoa and Koudougou areas), has led to massive immigration of seasonal agricultural labour, mainly originating from Burkina Faso (Mossi, Lobi ethnic groups), Mali (Malinke) and from the north (Senoufo) and the centre (Baoule) of Côte d'Ivoire [16,17]. The native Gouro people gradually gave their lands to the immigrants, usually the parcels situated between their fields and the forest, in order to protect their crops from wild animals (monkeys, elephants). The northern rural area has thus been exploited much earlier than the southern rural area, where there was no established village and only forest.

#### Landscape analysis

**Figure 4:**
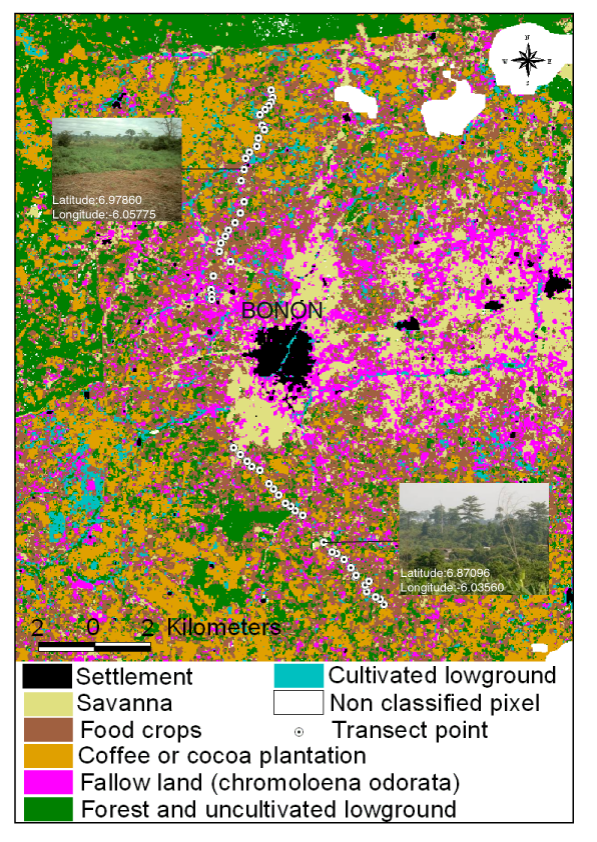
**Space structure of study area**. On this figure are located the two transects on foot, two pictures of the northern and southern rural areas, and the results of remote sensing analysis. The North and the South transects start in the rural area and go towards the town of Bonon. The pictures of the northern area show that almost all the landscape is exploited by humans. The pictures from the southern area show an association of crops and "natural" vegetation. With the remote sensing analysis results, we can see that all the study area is very exploited by humans and that the relict forest and uncultivated lowgrounds subsist only in the Marahoué national park and in parts of the southern rural area. In the northern rural area, most of the lowgrounds are cultivated. Coffee and cocoa plantations are present everywhere in the rural area, but not close to the biggest human settlements (town and villages).

The departure point of north transect (going towards Bonon) is an encampment surrounded by cocoa and coffee cultivation (figure 4). From there, the landscape opens up, the vegetation becomes lower and less dense and the land becomes flat. Some remnant big trees like the Samba (*Triplochiton scleroxylon*), the Iroko (*Chlorophora excelsa*), the Wara (*Kola gigantea*) and the kapok tree (*Ceiba pentandra*) testify to the earlier presence of mesophil forest. The transect continues through a succession of fallow burn and fallow with *Chromoloena odorata *(commonly called « Sékou Touré ») and finishes amongst cashew plantations, banana and manioc cultivation. At the northern periphery of Bonon town, the rice cultivation dominates the low-lying areas. The start of the south transect (going towards Bonon) is in a hilly plantation of coffee mixed with manioc. The line then crosses a small tributary with cocoa and banana trees, which turns into uncultivated lowlands characterized by the presence of bamboo. The principal cultivation is cash and food crops. The vegetation then becomes dominated by very dense herbaceous and shrub layers, which is flowed by a patch of relict forest, with *Kola cordifolia*, *Celtis zincheri *and *Ceiba pentandra*, through which free passage is prevented by a backwater and dense stands of *Acacia capensis*. Beyond the forest relict, the landscape is dominated by coffee and cocoa plantations. At the end of the transect, approaching the southern periphery of Bonon, the altitude decreases progressively, and the landscape becomes an herbaceous savannah. The major differences between the two transects are therefore the absence of forest and uncultivated low ground along northern transect, and the limited amount of cultivation along the southern line (pictures of figure 4). Analysis of LANDSAT imagery from 2000 shows that this pattern may be extrapolated to the whole study area (figure 4). This shows that, around the town and the villages located along the main road to the east, the original forest environment has been replaced by savannah, *Chromoloena odorata *fallow and food crops. To the north and west, is the boundary of the Marahoué National Park, which now constitutes a pioneer front for the coffee and cocoa plantations which cover much the landscape, in the north rural area as well as in the south area.

#### Human mobility

**Figure 5:**
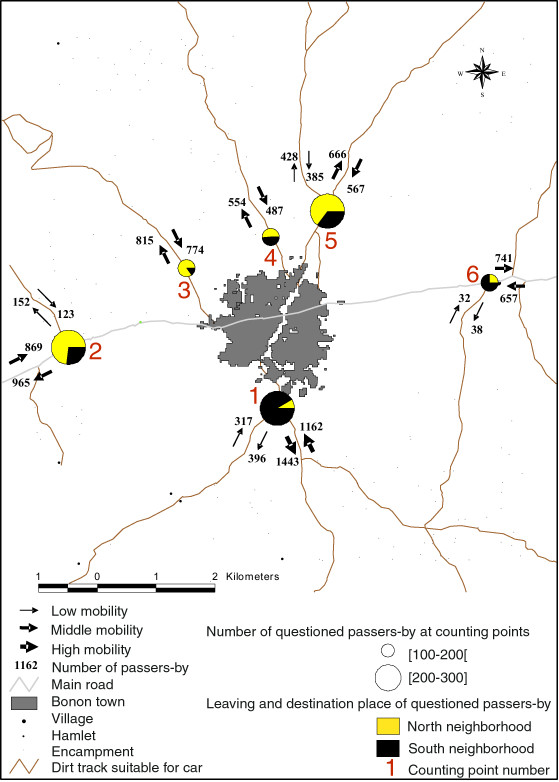
**Mean of daily human mobility between Bonon town and rural space, leaving and destination places of questioned passers-by**. This map shows the location of counting points and the results of the human mobility study. The counting points are located around Bonon, on the principal tracks which connect the town and the rural areas. We can see that the mobility in the study area is very important. The south/east track has the highest frequentation and most of the people who take this track live in the south neighborhoods of Bonon or in the southern rural area. We can also see that most of the people who take the northern tracks live in the north neighborhoods of Bonon or in the north rural area.

**Figure 6:**
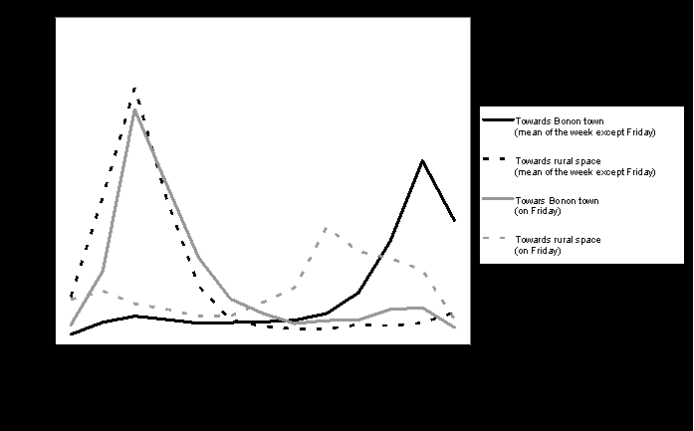
**Mobility between Bonon town and rural space according to direction and hour (mean of the week and Friday)**. This graph shows the characteristics (according to direction and hour) of mobility between Bonon and the rural area. During the week, people go to work on the morning in the rural area, except Friday (market day), where it is the opposite. The urban mobility is important during the week, but on Friday it is inverted due to the rural people who go to the town of Bonon to sell their products.

A total of 81,013 passers-by were counted at the 6 enumeration points (figure 5) over the course of a week, which demonstrates extreme mobility (there are 30,000 people living in the study area, 20,000 in Bonon town and 10,000 in the rural area). Of these, 37,619 were going from the rural area to Bonon and 43,394 from Bonon to the rural area. Out of the 81,013 people counted, 32,739 were in the north rural area (counting points 3, 4 and 5), 23,226 in the south rural area (counting point 1), 14,767 in the west rural area (counting point 2), and 10,281 in the east rural area (counting point 6). Figure 5 shows the mean mobility during the week of counting, for the different tracks and according to the direction taken. In the northern rural area movement is concentrated on three principal tracks. To the south of Bonon, the only track which goes to the south rural area is very heavily used (23,226 people). It is likely people counted one-way were probably counted again on their return; indeed, it was very clear throughout that in the morning, people were going from Bonon to the rural area, and in the evening people were coming back to the town. In figure 6 all observations made are aggregated according to slot time which shows that during the week, the urban population goes to the rural area in the morning and returns to Bonon in the evening, except on Friday (day of market in Bonon) when the rural population goes to town in the morning, and returns to the rural area in the evening. During the week, 1,174 people were questioned at the six counting points. Analysis of the answers shows that some of these people come from or go to other urban centers, mainly Bouaflé town, which seems to have an important link to the Bonon area. Particularly significant is the finding that people going to the northern rural area come mainly from northern neighborhoods, and people going to the southern rural area come mainly from southern neighbourhoods. Movement between rural areas (north, east, west, south) is thus very limited (figure 5) though the urban population is highly mobile.

## Discussion

In this study, we have used information about parasitological, entomological, human and environmental factors, to describe and explain the substantial spatial disparity of HAT between the southern and the northern sectors of the Bonon focus. The results help explain the spatial evolution of HAT prevalence in the Bonon focus since the 1950s. The entomological study undertaken here has shown that in the southern rural area, the density of tsetse flies (*Glossina palpalis palpalis*) is much higher than in the northern rural area. The places of transmission are apparently located in the southern rural area, mainly in the south-eastern area, frequented by many rural and urban patients, and where numerous tsetse flies (among which 2.67% are infected by *T. brucei*) were caught. The history of the area's development and its impact on the landscape may be related to the differences in vector density observed between the north and the south rural area. We have shown that at the beginning of the 20th century, the colonial government displaced the inhabitants of the north rural area towards the main road, and also that the south rural was uninhabited. Consequently, the north rural area has been exploited much earlier than the south rural area. The first waves of immigration were into the north area, where immigrants were settled near existing fields. The oldest and biggest settlement in the southern area, the Baoule hamlet called « Deux Côtes », was only created in 1977. In the southern area, contrary to the north, patches of both relict forest and uncultivated lowland remain, being favourable breeding areas for tsetse flies. The risk of trypanosome transmission is not, however, absolutely correlated to vector density, but depends also on a number of contextual factors [18,19] including human mobility. The track which goes to the south-east of Bonon is the most used, and goes through an environment favourable to the human-vector contact (edge of uncultivated lowland/plantations, edge of forest/communication routes) [20]. The transmission of HAT in this area is thus likely to be due to the high density of tsetse flies combined with a high human attendance, in an environment favourable to the human-vector contact. The lack of movement between the north and south is likely to be the main reason to explain that the disease spreads from the south towards the town, but does not spread onward into the densely inhabited northern rural area [8]. This area is thus characterized by a very low presence of vector and parasite, in an environment unfavorable to the human-vector contact, and the chance of transmission is thus too low to enable the development of HAT.

## Conclusion

In the focus of Bonon, the history of human settlement has apparently determined to a great extent, both current land cover patterns and the prevailing density of tsetse flies which in turn explain the spatial distribution of HAT. This sequence of events is likely to have happened in other foci within Côte d'Ivoire. Other factors remain, that could not be included in this study but which could also play an important role, such as the presence/absence of reservoir animals, feeding habits of tsetse, the evolution of landuse regarding the socio-political events that have recently occurred in Côte d'Ivoire.

In common with other studies, this work highlights the value of a GIS analysis in the context of multidisciplinary approach, to gaining an understanding of the distribution and spatial dynamics of the disease, and the necessary conditions for effective prevention, notably in Africa [21]. This work, by locating the most active transmission area at the south-east of the focus of Bonon, and by identifying the area with few disease cases, also helps to delineate the boundaries of the focus of Bonon more accurately. Targeted actions of tsetse control starting from north of Bonon (with little chance of tsetse reinvasion due to very low densities) to south, and targeted additional medical surveys in the south will be proposed to the Ivoirian HAT program, in order to reduce sleeping sickness in this area. The methodology used in the present work may be used in other areas of Côte d'Ivoire with similar history and similar environment to predict areas of possible sleeping sickness development/extinction. However it is likely that the current socio-political conditions in Côte d'Ivoire will have an impact on the further spread of HAT, on local, national and even international scales, particularly if forced movements of populations continue to occur [22].

## Methods

### Study area, presentation and description

The town of Bonon (7°N-6°W) is located about sixty kilometers west of the political capital of the country, Yamoussoukro (figure 1), in the Gouro ethnic group area,. The climate is equatorial with an annual rainfall of around 1 200 mm, and slight annual variations of temperatures (3°C). This region of the Central West Côte d'Ivoire is known for its historical (Bouaflé, Daloa) and contemporary (Vavoua, Sinfra) foci of sleeping sickness, but the focus of Bonon is comparatively recent [13-23]. The town of Bonon was created during the French colonial administration, when populations were forced to settle along communication routes, in order to have direct access to the labour necessary to the development, the maintenance and the exploitation of the area. In the early 70's, Bonon was a big village of agricultural labour immigrants, it has now become a commercial and administrative town (sub-prefecture of Marahoué region) of about 20 000 inhabitants. It is surrounded by villages, hamlets and encampments (with an additional total of about 10 000 inhabitants), representing more than fifty ethnic groups. Due to its infrastructure, Bonon has an important influence in nearby rural areas since the nearest urban centers (Daloa, Bouaflé, Sinfra) are located more than 30 kilometers away.

### Entomological survey

A total of 290 Vavoua traps [24] were set up on each patient's place of residence, water supply sites and working places- with the aim of obtaining data on the vectors distributions in relation human presence. Apparent Density per Trap per day (ADT: number of tsetse flies caught per trap and per day), infection by *Trypanosoma brucei *(the pathogenic parasite) using molecular tools (see below), and number of bloodmeals taken from humans were monitored. In the northern part (almost free of disease), traps were set up in areas the patients frequented. Each trap remained in place for four days, with cages being changed daily, and fly counts, sex-ratio determinations, and dissections being carried out daily. Tsetse flies (*G. palpalis*) were dissected to look for trypanosomes in the mouthparts, midgut, and salivary glands. Each organ (mouthparts, salivary glands, midgut) was put into a separate eppendorf tube containing 30 μl sterile distilled water for subsequent molecular analyses. *Trypanosoma brucei *identification was done by molecular analysis (PCR, polymerase Chain Reaction) using specific primers [25]. In order to assess levels of contact between human and tsetse, the bloodmeal origin was assessed using the method of Diallo *et al. *[26]. This method, based on variation of the Super Oxyde Dismutase (SOD) enzyme, distinguishes between bloodmeals taken from humans and animals.

### Geographical survey

#### Historical settlement of study area

Five administrative workers and several community leaders (religion, ethnic groups, youth) of town of Bonon and villages were interviewed, alternatively using individual or collective (around 20 people) discussions, in order to understand the history of the progressive settlement in this area. These interviews took place after several preliminary meetings in which the aim of the work was explained, in order to avoid any confusion due to the special context which prevailed in Côte d'Ivoire at the time of the study (March to May 2004). An additional objective was also to understand the evolution of the relationships between the different communities.

#### Landscape analysis

Two transects were carried out on foot (figure 4), one in the northern rural area of Bonon, the other in the southern rural area. The lines of these two transects were defined according to several parameters:

-Analysis of satellite image (Landsat 2000) covering the study area, which was used to determined landscape discontinuity.

-Knowledge of the area, acquired during the multiple travels by car and motorcycle.

-Where the patients lived.

These two transects were each 7 kilometers long. Landscape characteristics were assessed at points 250 metres apart using a description form, on which Global Positioning System (GPS) coordinates, landscape details, principal cultivation types and the names of the principal plant species were recorded. The data obtained were used to draw up a thematic classification of landscape, which was then extrapolated to the whole study area by the signal processing of Landsat 2000 satellite image. This signal processing was done under ENVI 3.2 (Environment for Visualizing images) software.

#### Human mobility

We defined six counting points (figure 5) on the principal tracks which link Bonon to the surrounding rural areas. At each of these points, every individual going from Bonon to the rural area and from the rural area to Bonon, from 6 am to 7 pm, every day during one week was recorded. At the same time, a random subsample was asked to complete a questionnaire, in order to obtain more detailed information on the nature of their journeys (place of departure, place of destination, reason for travelling).

All the data were recorded in a linked data base under Access. The data were then imported into Arcview 3.2., which was used to execute the spatial queries.

## Authors' contributions

Conception of the study: PS, JPD, VJ, FC.

Data acquisition, analysis, interpretation: FC, EO, BC, YO, VJ, SD, PS.

Critical revision of the manuscript: PS, VJ, GC, JPD.

All authors read and approved the final manuscript.
